# Participation of nurses and allied health professionals in research activities: a survey in an academic tertiary pediatric hospital

**DOI:** 10.1186/s12912-022-00922-1

**Published:** 2022-06-21

**Authors:** Matteo Amicucci, Immacolata Dall’Oglio, Valentina Biagioli, Orsola Gawronski, Simone Piga, Riccardo Ricci, Anna Angelaccio, Domenica Elia, Mario E. Fiorito, Luigi Marotta, Massimiliano Raponi, Emanuela Tiozzo, Research Study Group, Patrizia Amadio, Matilde Brancaccio, Ilaria Campagna, Gaetano Ciliento, Federica Connola, Matteo D’Angelo, Davide Della Lena, Michela Di Furia, Floriana Di Iorio, Giuliana Evangelisti, Rita Frezza, Daniele Gargano, Marjola Gjergji, Ciro Iorio, Antonella Lorubbio, Giulia Manzi, Rachele Mascolo, Francesca Maria Meloni, Flaminia Passi, Federico Piccioni, Angela Ragni, Desiree Rubei, Luisa Russo, Emanuela Salama, Gianna Scarselletta, Natalia Bianchi, Giuliana D’Elpidio, Marcello De Santis, Italo Ciaralli, Luisa Cirulli, Marina D’Agostino, Giovanna Manca, Sandra Martino, Mauro Paliotta, Anna Portanova, Alessandra Querciati, Tommaso Renzetti, Marco Roberti

**Affiliations:** 1 Department of Onco Haematology and Cell and Gene Therapy, Bambino Gesù Children’s Hospital, IRCCS, Rome, Italy; 2 Professional Development, Continuing Education and Research Service, Bambino Gesù Children’s Hospital, IRCCS, Sant’Onofrio Square 4, 00165 Rome, Italy; 3 Unit of Epidemiology, Bambino Gesù Children’s Hospital, IRCCS, Rome, Italy; 4 Department of Diagnostic Medicine and Laboratories, Bambino Gesù Children’s Hospital, IRCCS, Rome, Italy; 5 Department of Pediatric Subspecialties, Bambino Gesù Children’s Hospital, IRCCS, Rome, Italy; 6 Department of Images Diagnostic, Bambino Gesù Children’s Hospital, IRCCS, Rome, Italy; 7 Department of Intensive Neurorehabilitation and Robotics, Bambino Gesù Children’s Hospital, IRCCS, Rome, Italy; 8 Medical Directorate, Bambino Gesù Children’s Hospital, IRCCS, Rome, Italy

**Keywords:** Research, Nurses, AHPs, pediatric hospital, evidence-base nursing

## Abstract

**Background:**

Involvement in research activities is complex in pediatric nursing and allied health professionals (AHPs). It is important to understand which individual factors are associated with it to inform policy makers in promoting research.

**Methods:**

A cross-sectional observational study was conducted to describe the level of participation in research activities over the last ten years of nurses and AHPs working in a tertiary pediatric hospital. A large sample of nurses and AHPs working in an Italian academic tertiary pediatric hospital completed an online self-report questionnaire between June and December 2018. Three multivariate logistic regression analyses were performed to predict participation in research projects, speaking at conferences, and writing scientific articles.

**Results:**

Overall, data from 921 health professionals were analyzed (response rate = 66%), of which about 21% (*n* = 196) reported participating in a research project, while 33% (*n* = 297) had attended a scientific conference as a speaker, and 11% (*n* = 94) had written at least one scientific paper. Having a Master or a Regional Advanced Course, working as an AHP or a ward manager, as well as regularly reading scientific journals and participation in an internal hospital research group or attendance in a specific course about research in the hospital, significantly predicted participation in research projects, speaking at conferences and writing scientific papers. It is important to foster research interest and competencies among health professionals to improve participation in research projects, speaking at conferences, and writing scientific papers.

**Conclusions:**

Overall, we found a good level of attendance at conferences as speakers (33%), a moderate level of participation in research (21%), and low levels for writing scientific papers (11%). Our study highlighted the need to support participation in research activities among nurses and AHPs. Policymakers should identify strategies to promote research among nurses and AHPs, such as protected rewarded time for research, specific education, strengthened collaboration with academics, and financial support. Moreover, hospital managers should promote the development of research culture among health professionals, to improve their research competencies and evidence-based practice.

**Supplementary Information:**

The online version contains supplementary material available at 10.1186/s12912-022-00922-1.

## Background

The International Council of Nurses (ICN) has underlined the importance of health research both in academic and clinical settings for many years [1, 2]. Research plays a key role for nurses and allied health professionals (AHPs), including Rehabilitation Health Professionals, Health Technician Professionals, Midwives, Health Professionals of Prevention, in improving their professional development and promoting health innovation [3]. Overall, the roles and responsibilities of nurses and AHPs evolve and research values and enhances this evolution [4]. Therefore, research should underpin clinical practice in every health institution. On the one hand, research should be the mission of any academic institution and research hospital that fosters excellence and supports the development of health research activities [5]. These, even if only in part, have fostered the development of research activities for nurses and AHPs. On the other hand, nursing and AHP research has been more developed in English-speaking countries [6], where nurses and AHP researchers and academics have been conducting research by following a programmatic approach, as well as multicenter and multidisciplinary projects [7–9].

In order to improve evidence-based patient care, it is important to promote the culture of research among health professionals, including nurses and AHPs [10]. Conducting research does not only mean participating in research projects but also attending conferences and publishing scientific papers in peer-reviewed journals [11]. However, few studies propose efficient strategies that foster participation in research and are moreover difficult to put into practice in specific contexts. These strategies include constant updating, specific courses on research, reading scientific papers, and attending Journal Clubs [2–12]. The most virtuous institutions, the ones that make research their mission, endeavor to overcome barriers and put strategies into practice to increase research among nurses and AHPs.

Although nurses and AHPs are increasingly involved in research, only few studies describe how nurses and AHPs participate or are involved in research activities in the hospital setting. Many studies investigate and describe the barriers that hinder research conduction and utilization [13–19]. However, there is a need to conduct high quality studies aimed at producing and disseminating robust scientific evidence [20, 21]. In addition, it is important to have an overview of the level of involvement in research activities of nurses and AHPs and to understand which individual factors are associated with it to inform policy makers in promoting research.

Therefore, the aims of this study are: 1) to describe the level of participation in research activities in terms of participation in research projects, speaking at conferences, and writing scientific papers, over the last ten years in a sample of nurses and AHPs working in an Italian academic tertiary pediatric hospital; and 2) examine which socio-demographic and professional characteristics of nurses and AHPs are associated with these tasks.

## Methods

### Design

This is a cross-sectional observational study conducted between June and December 2018 through an online survey designed for a universal sample of nurses and AHPs working in an academic, tertiary, research pediatric hospital in Italy.

### Participants and setting

The study involved every nurse and AHP working in all of the five settings of the pediatric hospital: the main building; the research laboratories and outpatients’ clinic; the neurorehabilitation center; the sub-intensive neurorehabilitation, specialist medical and surgical center; and a branch in another region. The pediatric hospital has a total of 607 beds, 28,754 admissions or in rehabilitation (*n* = 605) per year, and employs 1300 nurses or pediatric nurses, and 300 AHPs. A “Nursing and AHP Research Unit” was established in 2009 with the purpose to enhance research activities among health professionals. Moreover, a research group was set up in 2008.

The inclusion criteria included being a staff, manager, or research nurse or AHP on duty in the study period in any clinical setting of the hospital where the study was conducted. The exclusion criteria were being a student or an attending professional, being on leave for sickness, maternity, or any other reason. The potentially eligible population included 1400 health professionals.

### Instruments

For the purpose of the study, we adapted a questionnaire we had previously developed for a similar research [2]. We introduced some new items to explore which courses in the field of research had been attended, participation in research activities, facilitating factors, and suggestions to improve participation in research activities. Before administering it to the study sample, the questionnaire was evaluated by a group of nurses and AHPs to assess its face and content validity. Experts found it easy to understand and exhaustive. The final version of the questionnaire consisted of 47 items, divided into 4 domains: 1) socio-demographic and occupational characteristics (e.g., gender, age, education, professional role); 2) educational activities in the field of research (e.g., reading scientific journals, specific courses on research, attending periodic meetings within the hospital); 3) participation in research projects (active participation in research through project planning or data collection in research studies); 4) speaking at conferences (active participation in national or international conferences as invited speaker or with an abstract accepted as oral communication); and 5) writing scientific papers (author of an article in Italian or English published in a peer-reviewed journal).

The survey refers to respondents’ research activities in the previous 10 years, between the beginning of 2008 and the end of 2017. The average estimated amount of time required to complete the survey was about 20 min.

### Data collection

Data were collected between June and December 2018. The final questionnaire was launched through *Survey Monkey*® (a software for online surveys). The researchers and the members of the internal hospital research group presented the study to all nurses and AHPs during dedicated meetings held in the clinical units during the months of June, July, and August 2018, to inform them about the purpose of the study and to motivate them to participate. In addition, a flyer was produced to spread the information about this study. The researchers sent via email the invitation to potential study participants, including information about the purpose of the survey, and the link to the online survey. Participants were invited to complete the survey within 30 days. Those who had not completed the survey within the deadline received a reminder via email.

### Ethical considerations

The Ethics Committee of the hospital approved this study (Registration number: 1525_OPBG_2018]. The nurses and the AHPs were informed about the objectives and how to participate in the study. At the beginning of the online survey, participants were asked to sign an informed consent for their participation, which was required to access the online survey. Participants unable to complete the survey online, were provided with the paper and pencil version of the questionnaire. Participants were asked to indicate their full name, therefore data were not anonymous. This was necessary to monitor their participation in research activities both retrospectively and prospectively.

### Data analysis

Categorical variables were summarized using absolute frequencies and percentages, and continuous variables by the mean or median and interquartile ranges, as appropriate. To determine statistical differences between groups, we used the Chi-square test or Fisher’s exact test for categorical variables, and the t-test or Mann–Whitney test for continuous variables. Three multivariate logistic regression analyses were tested to identify explanatory variables of the three primary study outcomes: participation in research projects, speaking at conferences, and writing scientific papers. At first, in the multivariate models we included all the variables with P < 0.20 following univariate analyses and then we restricted the model by selecting only significant variables at regression. Final models were computed with a stepwise backward procedure (likelihood ratio test, *P* < 0.05). All statistical analyses were performed using STATA, Statistical Software: Release 13 (StataCorp LP, College Station, TX).

### Validity, reliability, and rigor

Although the questionnaire we used was not psychometrically tested, it had been previously administered for a similar study and found useful and acceptable [22]. Moreover, a consensus‐based approach with a group of nurses and AHPs was carried out to confirm each item. Content evaluation of the questionnaire involved numerous iterations, until consensus was achieved on the wording and format of each item. Pilot testing took place before the beginning of the survey. Although data were not collected anonymously, participants were assured that information would be used for research purposes only. The analyses were carried out by a blinded evaluator.

## Results

### Sample characteristics

Out of 1,400 eligible healthcare professionals, about 1,300 e-mails including the link to the survey were sent. Overall, data from 921 questionnaires were analyzed after removing duplicate responses. The response rate was 66% (63.5% for nurses and 79.5% for AHPs). The demographic, professional and education characteristics of participants are shown in Table 1. Most of the participants were female (82%), with a mean age of 40.66 years (SD = 11.73). Participants were pediatric nurses (42.4%), nurses (40.8%) and AHPs (16.7%), such as laboratory technicians (5.2%), radiology technicians (2.2%), neuropsychomotricity therapists (2.1%), speech therapists (1.4%), neurophysiopathologist technicians (1.3%), physiotherapists (1.1%), dietitians (1.1%), and other health professionals (2.3%). About 7% had a manager position in a unit or in a department or other services (1.4%). The mean length of work experience was 15.55 (SD = 12.71) years. More than one third reported previous working experience outside the hospital where the study was conducted; 18.6% of these concerned pediatric care. Regarding the educational background, the Bachelor’s Degree was the most frequent title (58.3%), while about 27% also had a Regional Diploma. About 14% of the respondents had post registration education titles, such as a Master’s in Management, and about 12% had a Master of Science in Nursing. Almost 50% of the healthcare professionals had postgraduate clinical education (Master or specialization courses) (Table 1).

**Table 1:** Demographic, professional and education characteristics (*n* = 921)

	**n**	**%**
Gender		
Females	756	82.0
Males	165	18.0
Age in years		
22–29	212	23.0
30–39	258	28.1
40–49	178	19.3
≥ 50	273	29.6
Professional qualification		
Registered nurse	376	40.8
Registered paediatric nurse	391	42.5
Allied Health Professional	154	16.7
Professional role		
Staff	806	87.5
Manager	79	8.5
Clinical expert	15	1.7
Fellow	15	1.7
Other	6	0.6
Work experience in hospital (years)		
≤ 4	235	25.5
5–9	153	16.6
10–19	222	24.1
20–29	97	10.5
≥ 30	214	23.2
Hospital employee		
Yes	774	84.0
Hospital center		
Main building	616	66.9
Research laboratories and outpatients	82	8.9
Sub-intensive neurorehabilitation, specialist medical and surgical	197	21.4
Neurorehabilitation	25	2.7
Branch in another region	1	0.1
Previous work outside hospital		
Yes	354	38.4
Education level		
Regional Diploma (RD)	245	26.6
University Diploma (UD)	48	5.2
Bachelor’s Degree (BD)	537	58.3
RD + UD/BD	54	5.9
UD + BD	37	4.0
Education for manager roles (*n* = 176)		
Regional qualifying course for manager roles	49	5.3
Master in Management	127	13.8
Education for executive roles (*n* = 130)		
Master of Science in Nursing	110	11.9
Director of Nursing Services	10	1.1
Both	10	1.1
Postgraduate clinical education (*n* = 462)		
Master^a^	247	26.8
Advanced Master^b^	8	0.87
Specialization course	124	13.7
University improvement courses	78	8.47
PhD (or PhD student)	5	0.5
Participation in hospital research group (*n* = 850)	136	16
Participation in at least one specific course about research in hospital (*n* = 850)	113	13.3
Participation in other courses on research outside the hospital (*n* = 850)	46	5.41

^a^A Post-graduate Diploma (after a Bachelor’s Degree)

^b^A Post-graduate Diploma (after a Master’s Degree)

### Reading journals and level of knowledge of epidemiology, statistics, and English

Most healthcare professionals reported reading journals (79.7%), either routinely (12.8%), or occasionally (32.5%) or when looking for specific issues (34.3%). Most read Italian journals only (61.1%), while 32.2% also read international journals (Table 2). Moreover, ‘good’ or ‘very good’ knowledge of epidemiology, statistics and English were reported by 13.9%, 8.2% and 18.3% of the respondents, respectively (Table 2). In particular, English reading skills were rated higher than writing (good or very good ratings: 32.8% versus 20.8%).

**Table 2:** Reading scientific journals and knowledge of epidemiology, statistics, and English

	**n**	**%**
Reading journals (*n* = 865)		
No	169	19.5
Yes, usually	111	12.8
Yes, occasionally	281	32.5
When I look for something	297	34.3
Other	7	0.8
Type of journal (n = 882)		
Italian only	539	61.1
International	59	6.6
Italian and International	284	32.2
Number of journals (*n* = 678)		
One	354	52.2
Two	178	26.3
Three or more	146	21.5
Knowledge of Epidemiology (*n* = 897)		
Very good	10	1.1
Good	115	12.8
Fairly good	193	21.5
Sufficient	293	32.7
Insufficient	185	20.6
Poor	101	11.3
Knowledge of Statistics (*n* = 897)		
Very good	4	0.4
Good	70	7.8
Fairly good	177	19.7
Sufficient	282	31.4
Insufficient	232	25.9
Poor	132	14.7
Knowledge of English (*n* = 897)		
Very good	29	3.2
Good	135	15.1
Fairly good	187	20.8
Sufficient	274	30.5
Insufficient	181	20.2
Poor	91	10.1

### Level of participation in research projects, speaking at conferences, and writing scientific papers

In the period between 2008–2018, 193 (21.3%) participants reported participating in a research project, 297 (33.1%) attended a scientific conference as a speaker, and 94 (10.7%) had written a scientific paper (*n* = 94, 10.7%) (Supplemental Fig. 1). Of these 94 participants, only 10 (11%) provided the details of their publications.

Of the healthcare professionals, 37.8% had been involved in only one research project, 23.0% in two, and 39.3% in three or more. The themes of the projects were mainly: nursing (57.1%), medical (35.7%), technical (16.8%), rehabilitation (3.2%), other (7.6%). The study designs were qualitative (8.7%), quantitative (7.3%), and mixed (9.3%).

With regard to attending conferences as speakers, 163 (54.9%) healthcare professionals reported that they attended up to five conferences, 43 (14.5%) attended 6–10 conferences, and 89 (30%) > 11 conferences. During the same time, 77 health professionals published all or part of their talks either as conference proceedings (4.3%) or papers in journals (5.5.%). With regard to the number of published scientific papers: 45 (47.9%) healthcare professionals had published one paper, 18 (19.1%) two papers, 16 (17.0%) between 3–5 papers, and 15 (16.0%) more than 5 papers.

### Univariate associations between study variables

Factors significantly associated with higher research participation were male gender (*p* < 0.001), having a Bachelor’s Degree (*p*  < 0.001), having achieved a Master in Management (*p* < 0.001), a Master's Degree (*p*  < 0.001), a Master (*p*  < 0.001), an Advanced Master (*p* < 0.001) or a Regional Advanced Course (*p*  < 0.001), being an AHP rather than a nurse (*p*  < 0.001), being a manager (*p*  < 0.001), a clinical specialist (*p*  < 0.001), or a research fellow (*p*  < 0.001), and working in the main building of the hospital (*p*  = 0.013) or in the research laboratories and outpatients clinic (*p*  = 0.033) rather than in the sub-intensive neurorehabilitation, specialist medical and surgical center. In addition, other variables related to the interest in research were significantly associated with higher research participation (see Supplemental Table 1).

Factors significantly associated with the role of speaker at a scientific conference were male gender (*p*  = 0.049), age over 30 years (*p*  = 0.003), work experience longer than 5 years (*p*  = 0.047), having achieved a Master’s Degree alone (p < 0.001) or together with a course as director of nursing units (*p*  = 0.009), a Master (*p*  < 0.001), working as an AHP rather than a nurse (*p*  < 0.001), being hospital-employed (*p*  < 0.001), being a manager (*p*  < 0.001) or a clinical specialist (*p*  = 0.001), and working in the main building of the hospital (*p*  = 0.014). In addition, other variables related to the interest in research were significantly associated with speaking at conferences (see Supplemental Table 2).

Factors significantly associated with writing scientific articles were male gender (*p*  = 0.022), having a Bachelor’s Degree (*p*  = 0.006), a Master’s Degree (*p*  < 0.001), a Master in Management (*p*  = 0.001), a Regional Qualifying Course for manager roles (*p*  = 0.005), a Master (*p*  < 0.001), an Advanced Master (*p*  < 0.001) a Regional Advanced Course (*p*  < 0.001), or a PhD (*p*  < 0.001); working as an AHP rather than a nurse (*p*  < 0.001), being a manager (*p*  < 0.001) as clinical specialist (*p*  = 0.023) or research fellow (*p*  = 0.001), working in the main building of the hospital (*p*  = 0.020) or in research laboratories and outpatients clinic (*p*  = 0.001). In addition, other variables related to interest in research were significantly associated with writing scientific papers (see Supplemental Table 3).

### Multivariate associations between study variables

**Fig. 1:**
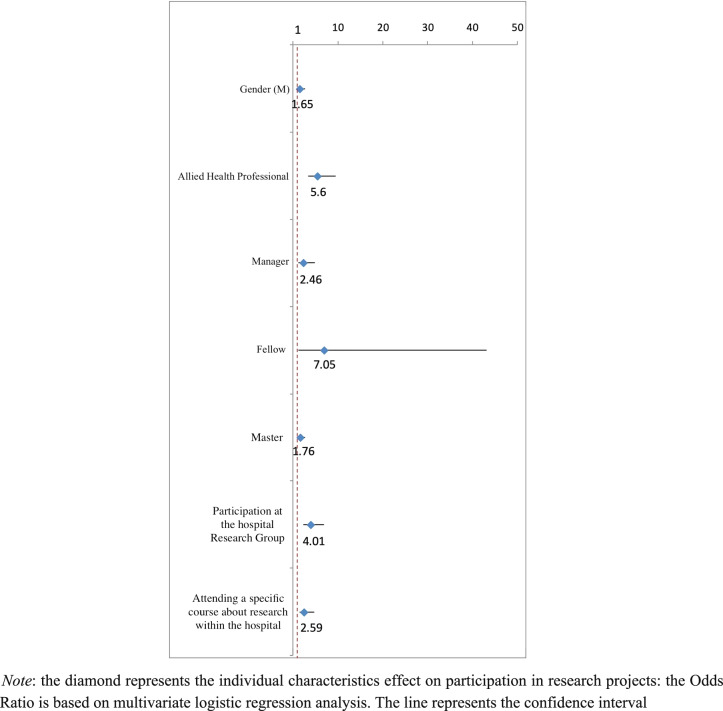
Forest Plot of the characteristics of the sample associated with participation in research projects based on multivariate regression analysis

Univariate associations between study variables were investigated. Regression analysis confirmed that factors independently associated with higher research participation included working as an AHP (*p*  < 0.001), having a role as unit manager (*p*  = 0.011), having a Master (*p*  = 0.014), and attending a specific course about research within the hospital (*p*  = 0.002) (Fig. 1, Supplemental Table 1). Other factors were male gender (*p*  = 0.049), being a research fellow (*p*  = 0.035), and participation at the internal hospital research group (*p*  < 0.001).

**Fig. 2:**
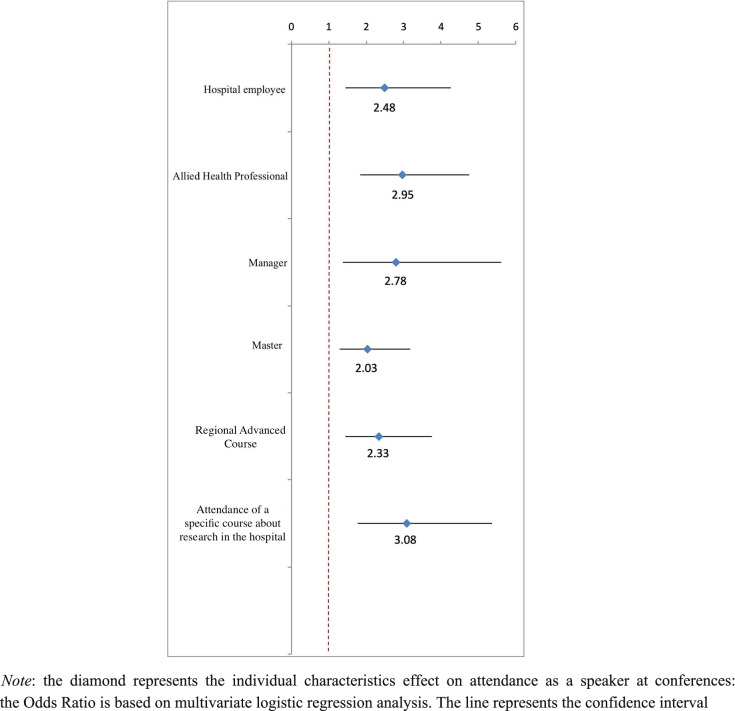
Forest Plot of the characteristics of the sample associated with attendance as a speaker at conferences based on multivariate regression analysis

Regression analysis confirmed that factors independently associated with speaking at a scientific conference included working as an AHP (*p*  < 0.001), being a manager (*p*  = 0.004), being hospital-employed (*p*  = 0.001), having a Master (p = 0.002) or a Regional Advanced Course (*p*  < 0.001), and attendance of a specific course about research in the hospital (*p*  < 0.001) (Fig. 2, Supplemental Table 2).

**Fig. 3:**
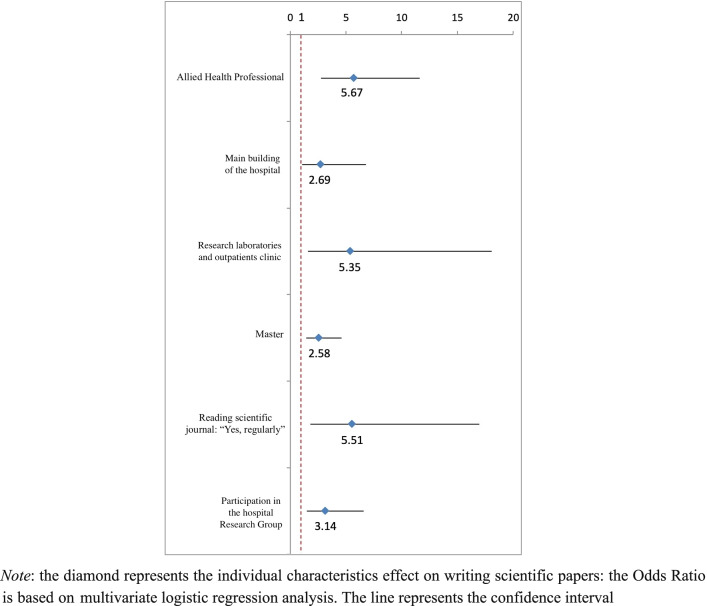
Forest Plot of the characteristics of the sample associated with writing scientific papers based on multivariate regression analysis

The explanatory variables significantly associated with writing scientific articles included working as an AHP (*p*  < 0.001), in the main building of the hospital (*p*  = 0.036) or in research laboratories and outpatients clinic (*p*  = 0.007), having a Master (*p*  = 0.002), regularly reading scientific journals (*p*  = 0.003), and participation in the internal hospital research group (*p*  = 0.002) (Fig. 3, Supplemental Table 3).

## Discussion

This is one of the first studies that describes the level of participation in research in both nurses and AHPs working in an academic tertiary pediatric hospital. Overall, we found a good level of attendance at conferences as speakers (33%), a moderate level of participation in research (21%), and low levels for writing scientific papers (11%). In particular, the level of participation in research projects was similar to other studies conducted with pediatric or general nurses [23, 24] but lower than that reported for health professionals [25, 26].

Despite this was a monocentric study, the large number of survey participants, larger than in other similar studies including nurses or AHPs [27–29], corroborates its results. The survey response rate was higher also compared to studies investigating attitudes towards research reported by both nurses and AHPs, ranging from 7 to 24% [10, 30]. This could be an indicator of the nurses’ and AHPs’ interest in this survey. However, AHPs were more responsive and willing to participate in the study compared to nurses, maybe because nurse research participation was still not sufficiently recognized by the organization as a criterion for professional evaluation and career development. Moreover, this was the first time a similar study involved also AHPs in this hospital. Consequently, it is possible that participation in this study was considered a good opportunity for AHPs to give visibility to their scientific activities. This desire to be visible may also explain why working as AHP rather than a nurse was found to predict higher research participation, speaking at conferences, and writing scientific papers. Although this finding is in contrast with Luckson et al. (2018) [10], it is important to note that many roles considered as AHPs in Italy, in other countries are performed at advanced nursing practice levels. Another possible explanation is that the more experienced AHPs represented a role model in the field of research for their discipline so that the new generation of AHPs had the opportunity to benefit from them. Although nurses had developed a similar role-modeling process, it started more recently, and the results will probably be evident in the next few years. In addition, AHPs published scientific papers not only in their own area but also in the medical area [31].

Overall, our results showed that the number of nurses writing scientific papers was suboptimal. In Ireland, the number of nurses who had published a scientific paper was higher (17.5%) already back in 2008 [31], therefore this aspect needs to be improved in Italy. However, it is possible that the number of nurses and AHPs involved in scientific writing in other non-English speaking countries is also suboptimal [30]. For Italian nurses and AHPs, the English language is probably the most significant barrier that prevents them from reading international journals and writing scientific papers. In fact, only a small number of participants reported to have an excellent/good knowledge of English. This may explain why few nurses and AHPs read international journals. Similar results were obtained also from other studies conducted in non-English speaking countries, like Finland [32] or Turkey [29]. Other barriers described in the literature include lack of time, confidence or knowledge about research, and organizational support [23]. These kinds of barriers could explain the low level of involvement in research projects, which we observed also in our academic tertiary and research hospital. Further studies are needed to investigate the impact of language barriers on research. National institutions and the international community might work together to provide additional resources, mentoring, and support for nurses and AHPs in hospitals, especially in countries with limited resources.

Although suboptimal, speaking at conferences was the most frequent research activity reported by participants. This may be due to nurses’ and AHPs’ wish to share their professional experiences at conferences, which constitutes a first step in approaching the scientific community. This is particularly true for managers, who may have a greater interest in these events and benefit from organizational support provided by their hospital. Being the manager of a hospital unit or a department was also found to predict higher participation in research. This was an expected result, because managers are often involved in research due to their leading positions in the hospital, their knowledge of the healthcare processes, their constant presence, and their support in data collection. However, their important research activity is rarely reflected in their scientific publications, as also reported by other studies [20, 33]. This may be due to their low awareness of the importance of publishing scientific papers and their relatively low professional benefit from being an author. In fact, authorship in scientific papers is still not mandatory for managers’ professional development, considering also that scientific output is generally not one of the evaluation criteria for these roles in the institution.

We found that having one or more postgraduate academic titles, such as a Master, positively predicted higher participation in research projects, speaking at conferences and writing scientific papers. Professionals who have more advanced levels of education tend to be more motivated and willing to address research problems that arise from clinical practice and from their own clinical experience, since they are more knowledgeable about research methods [34]. Moreover, university students are often required to be involved in research projects for their final thesis [29]. This underlines the importance of strengthening the academic exchanges with hospitals to better integrate research competencies into clinical practice and improve the level of participation in research activities among health professionals [35]. Moreover, although doctoral programs (PhDs) are available for nurses and AHPs in Italy [12], only few participants reported having achieved this title. Therefore, it is paramount in the future to facilitate and support the participation of nurses and AHPs in PhD programs, and ensure sufficient teaching and mentoring to a larger number of health professionals.

Interestingly, male gender was found to predict significantly higher participation in research. This may indicate that male AHPs have greater interest and/or the availability of being involved in research and pursuing research opportunities and careers. Reasons for this “gender gap” include women’s limited time to invest in research because of family obligations, unequal representation of women in mentorship and leadership roles, whereby the different gender opportunities perception is perpetuated in newer generations, and it results in different career expectations [36–38]. However, the impact of not having women represented at a higher level may lead to a discriminative approach to research priorities/activities. Therefore, their participation is warranted and should be facilitated, for example by providing more and better support services for families.

In addition, participating in specific research courses or being part of the internal hospital research group proved to be one of the most important predictors for participation in research and writing scientific publications. This finding was also confirmed by two similar but non-specific pediatric studies [10, 30], underlying the importance of education and continuing education, as well as communication and dissemination of research findings. Being part of the internal hospital research group means taking part in monthly meetings on a research topics and Journal Clubs. In particular, Journal Clubs for both nurses and AHPs are a winning strategy to approach research [2, 3]. Moreover, Journal Clubs promote reading regularly scientific journals, which was identified as one of the most important predictive factors for the publication of scientific papers, in line with other authors [3]. Therefore, the improvement of this aspect could be a very important goal to achieve, especially if focused on international pediatric journals [14, 39].

### Limitations

The study population is limited to one hospital, therefore any generalization from the results must be made with caution. The questionnaire, as in other studies [39, 40], was specially designed for this survey and was not psychometrically tested. We considered that validated questionnaires present in literature on this issue [10, 30] were with many questions mostly focused on attitudes towards research but not about research activities. They were therefore not a perfect fit for the scope of our research and presented the risk that the nurses and AHPs would not answer at all. This might limit the possibility to compare the results of this study with those of other studies. Moreover, the non-anonymous approach used for the surveys may have resulted in social desirability bias. Although participants were asked to report their research experience in the last ten years, the cross-sectional study design could limit the possibility of monitoring research participation and scientific production of the participants in the long term.

## Conclusions

This study provided a snapshot of current research activities among the nurses and AHPs in an Italian academic tertiary pediatric hospital across its five centers. Nevertheless, the hospital is a research institute, the level of engagement in research of nurses and AHPs was still weak. New interventions need to be implemented to improve research participation and scientific production in the hospital. Firstly, research collaboration among different health professions should be facilitated. Moreover, collaboration between researchers and clinical practitioners, especially clinical specialists, need to be enhanced. Another point could be to strengthen academic exchange so that nurses and AHPs may learn by applying research methodologies. In addition, the development of national and international research networks between clinical centers should be fostered to increase research capacity. Finally, policy makers should identify strategies to promote the development of high-quality nursing and AHP research, such as protected rewarded time for research, specific education, strengthened collaboration with academics, and financial support [35].

## Supplementary Information

**Additional file 1: Supplemental Table 1.** Descriptive variables of thesample and association with participation in research projects: univariate andmultivariable logistic analyses.

**Additional file 2: Supplemental Table 2.** Descriptive variables of thesample and association with attendance as a speaker at conferences: univariateand multivariable logistic analyses.

**Additional file 3: Supplemental Table 3.** Descriptive variables of thesample and association with writing scientific papers: univariate andmultivariable logistic analysis.

**Additional file 4: Supplemental Figure 1.** The three main outcomes ofstudy research.

## Data Availability

The datasets used and/or analyzed during the current study are available from the corresponding author on reasonable request.

## Abbreviations

AHP: Allied Health Professional
ICN: International Council of Nurses
