# Supplementary material for: Participation of nurses and allied health professionals in research activities: a survey in an academic tertiary pediatric hospital
Source: BMC Nurs. 2022 Jun 21;21:159. doi: 10.1186/s12912-022-00922-1 (PMC9210609; doi:10.1186/s12912-022-00922-1)
Supplement: Supplementary file 1 — Additional file 1: Supplemental Table 1. Descriptive variables of thesample and association with participation in research projects: univariate andmultivariable logistic analyses. [file 12912_2022_922_MOESM1_ESM.pdf]

Supplemental Table 1. Descriptive variables of the sample and association with participation in research projects: univariate and multivariable logistic analyses

	Participation in research projects		Univariate analysis			Multivariate analysis		
	n	%	OR	95%CI	P-value	OR	95%CI	P-value
Gender								
Females	144	19.05	1	-				
Males	52	31.52	1.96	1.34-2.85	<0.001	1.65	1.00-2.73	0.049
Age in years								
22-29	46	21.70	1	-				
30-39	60	23.26	1.09	0.71-1.69	0.69			
40-49	37	20.79	0.95	0.58-1.54	0.83			
≥50	53	19.41	0.87	0.56-1.35	0.54			
Professional Qualification								
Registered Nurse	71	19.09	1	-				
Registered Pediatric Nurse	53	13.59	0.67	0.45-0.98	0.04	0.95	0.55-1.62	0.846
Allied Health Professional	72	45.28	3.51	2.34-5.26	<0.001	5.59	3.31-9.45	<0.001
Professional role								
Staff	163	19.38	1	-				
Manager	33	41.21	3.30	2.04-5.33	<0.001	2.46	1.23-4.92	0.011
Clinical expert	8	47.06	4.17	1.58-11.01	<0.001	1.01	0.26-3.92	0.989
Fellow	13	86.67	30.53	6.81-136.79	<0.001	7.05	1.15-43.16	0.035
Hospital employee								
No	33	22.45	1					
Yes	163	21.06	0.92	0.60-1.41	0.706			
Hospital center (n=920)								
Sub-intensive neurorehabilitation, specialist medical and surgical	29	14.72	1	-				
Main building	142	23.05	1.73	1.12-2.68	0.013			
Research laboratories and outpatients	21	25.61	1.99	1.06-3.76	0.033			
Neurorehabilitation	5	20.00	1.45	0.50-4.16	0.492			
Work experience in hospital (years)								
≤4	54	22.98	1	-				
5-9	35	22.88	0.99	0.61-1.61	0.98			
10-19	49	22.07	0.95	0.61-1.47	0.82			
20-29	18	18.56	0.76	0.42-1.38	0.38			
≥30	40	18.69	0.77	0.49-1.22	0.27			
Education level								
Other (Regional Diploma, University Diploma, etc.)	49	15.41	1					
Bachelor's Degree	149	24.71	1.80	1.26-2.57	<0.001	1.42	0.81-2.46	0.218
Education for manager roles								
None	140	18.79	1	-				
Regional qualifying course for manager roles	13	26.53	1.56	0.81-3.02	0.19			
Master in Management	43	33.86	2.21	1.47-3.34	<0.001			
Education for executive roles								
No	142	17.95	1	-				
Master of Science in Nursing	48	43.64	3.53	2.32-5.38	<0.001			
Director of Nursing Services	2	20.00	1.14	0.24-5.44	0.86			
Both	4	40.00	3.05	0.85-10.94	0.08			
Post-graduate education								
No	115	17.37	1	-				
Master [±]	74	29.96	2.03	1.45-2.85	<0.001	1.76	1.12-2.76	0.014
Advanced Master [†]	5	62.50	7.93	1.87-33.64	<0.001	1.41	0.16-12.14	0.752

Post-graduate Courses or Regional specializations								
No	150	19.21	1	-				
Yes	43	34.68	2.23	1.48-3.37	<0.001	1.67	0.93-3.01	0.086
PhD (or PhD student)	4	0.43	1	-	-	1	-	
Reading scientific journals (n=882)								
Frequency								
No	15	8.8	1	-				
Yes, occasionally	45	16.01	1.96	1.05-3.63	0.03			
Yes, when I have to search for something	61	20.54	2.65	1.46-4.84	<0.001			
Yes, regularly	51	45.95	8.73	4.56-16.69	<0.001			
Types of journals								
International	59	10.95	1	-				
Italian	32	54.24	9.64	5.40-17.21	<0.001			
Both	86	30.28	0.37	0.21-0.65	<0.001			
Knowledge of Epidemiology (n=897)								
None/Insufficient	60	19.35	1	-				
Sufficient/Fair	92	18.93	0.97	0.68-1.40	0.880			
Excellent/Good	46	36.80	2.42	1.53-3.84	<0.001			
Knowledge of Statistics (n=897)								
None/Insufficient	63	16.24	1	-				
Sufficient/Fair	101	22.00	1.45	1.03-2.06	0.035			
Excellent/Good	34	45.95	4.38	2.58-7.46	<0.001			
Knowledge of English (n=897)								
None/Insufficient	43	19.35	1	-		1	-	
Sufficient/Fair	103	22.34	1.69	1.14-2.50	0.008	1.63	0.93-2.86	0.089
Excellent/Good	52	31.71	2.73	1.72-4.33	<0.001	1.90	0.95-3.80	0.071
Participation in hospital research group								
No	96	13.45	1	-				
Yes	72	52.94	7.24	4.86 - 10.80	<0.001	4.01	2.32-6.94	<0.001
Participation in at least one specific course on research in hospital								
No	105	14.25	1	-				
Yes	63	55.75	7.59	4.96 - 11.60	<0.001	2.59	1.43-4.69	0.002
Participation in other courses on research outside the hospital								
No	144	17.91	1					
Yes	24	52.17	5	2.73 - 9.16	<0.001	2.14	0.90-5.05	0.084

Note: ±A Post-graduate Diploma (after a Bachelor's Degree); †A Post-graduate Diploma (after a Master's Degree).
