# Supplementary material for: Participation of nurses and allied health professionals in research activities: a survey in an academic tertiary pediatric hospital
Source: BMC Nurs. 2022 Jun 21;21:159. doi: 10.1186/s12912-022-00922-1 (PMC9210609; doi:10.1186/s12912-022-00922-1)
Supplement: Supplementary file 2 — Additional file 2: Supplemental Table 2. Descriptive variables of thesample and association with attendance as a speaker at conferences: univariateand multivariable logistic analyses. [file 12912_2022_922_MOESM2_ESM.pdf]

Supplemental Table 2. Descriptive variables of the sample and association with attendance as a speaker at conferences: univariate and multivariable logistic analyses

	Speaking at a conference		Univariate analysis			Multivariate analysis		
	**n**	**%**	**OR**	**95%CI**	**P-value**	**OR**	**95%CI**	**P-value**
Gender								
Females	234	31.71	1	-				
Males	63	39.87	1.43	1.00-2.03	0.049	1.40	0.90-2.18	0.140
Age in years								
22-29	47	22.82	1	-				
30-39	89	35.60	1.87	1.23-2.83	0.003			
40-49	63	36.42	1.94	1.24-3.04	0.004			
≥50	98	36.70	1.96	1.30-2.95	0.001			
Professional qualification								
Registered Nurse	102	28.25	1	-				
Registered Paediatric Nurse	115	30.18	1.10	0.80-1.51	0.564	1.32	0.88-1.96	0.179
Allied Health Professional	80	51.95	2.74	1.86-4.06	<0.001	2.95	1.83-4.76	<0.001
Professional role								
Staff	230	29.11	1	-				
Manager	51	65.38	4.60	2.81-7.51	<0.001	2.78	1.38-5.60	0.004
Clinical expert	12	75.00	7.30	2.33-22.88	0.001	2.67	0.70-10.22	0.151
Fellow	4	33.33	1.22	0.36-4.08	0.750	0.67	0.15-3.09	0.612
Hospital employee								
No	27	19.15	1	-				
Yes	270	35.76	2.35	1.51-3.67	<0.001	2.48	1.44-4.27	0.001
Hospital center (n=920)								
Sub-intensive neurorehabilitation, specialist medical and surgical	142	73.96	1	-		1	-	
Main building	386	64.33	1.57	1.09-2.26	0.014	1.53	0.999-2.34	0.050
Research laboratories and outpatients	56	71.79	1.11	062-2.01	0.716	0.79	0.38-1.61	0.511
Neurorehabilitation	15	60.00	1.89	0.80-4.49	0.147	0.80	0.27-2.34	0.684
Work experience in hospital (years)								
≤4	56	24.56	1	-				
5-9	51	34.00	1.58	1.00-2.49	0.047			
10-19	76	35.51	1.69	1.12-2.55	0.012			
20-29	37	38.95	1.96	1.17-3.27	0.010			
≥30	77	36.84	1.79	1.19-2.70	0.006			
Education level (n=896)								
Other (Regional Diploma, University Diploma, etc.)	105	33.87	1	-				
Bachelor's Degree	192	32.76	0.95	0.71-1.27	0.738			
Education for manager roles								
None	206	28.41	1	-				
Regional qualifying course for manager roles (AFD)	27	57.45	0.30	0.17-0.54	<0.001	1.22	0.53-2.82	0.642
Master in Management	64	51.61	0.39	0.27-0.57	<0.001	0.98	0.53-1.81	0.955
Education for executive roles								
No	232	30.13	1	-				
Master of Science in Nursing (MSN)	55	51.40	2.45	1.63-3.69	<0.001	1.62	0.97-2.72	0.067
Director of Nursing Services (DAI)	3	30.00	0.99	0.25-3.88	0.993	0.59	0.09-3.97	0.586
both	7	77.78	8.11	1.67-39.36	0.009	4.09	0.61-27.29	0.146
Post-graduate education								
No	182	28.22	1	-				
1st Level 1-year Master Course±	108	45.19	2.10	1.54-2.85	<0.001	2.03	1.29-3.18	0.002
2nd Level 1-year Master Course†	4	50.00	0.88	0.22-3.62	0.866	1.11	0.15-7.97	0.915

Post-graduate Courses or Regional specializations								
No	225	29.61	1	-				
Yes	67	55.37	0.35	0.24-0.52	<0.001	2.33	1.45-3.75	<0.001
PhD (or PhD student)	4	0.43	1	-				
Reading scientific journals (n=882)								
Frequency								
No	38	22.49	1	-				
Yes, occasionally	79	28.11	1.35	0.86-2.10	0.188			
Yes, when I have to search for something	114	38.38	2.15	1.40-3.30	<0.001			
Yes, regularly	54	48.65	3.27	1.94-5.48	<0.001			
Types of journals								
Italian	134	24.86	1	-				
International	33	55.93	3.83	2.21-6.65	<0.001			
Both	122	42.96	2.28	0.27-0.40	<0.001			
Knowledge of Epidemiology (n=897)								
None/Insufficient	91	31.82	1	-				
Sufficient/Fair	159	32.78	1.04	0.76-1.43	0.782			
Excellent/Good	47	37.60	1.29	0.83-2.00	0.254			
Knowledge of Statistics (n=897)								
None/Insufficient	111	30.49	1	-				
Sufficient/Fair	151	32.97	1.12	0.83-1.51	0.450			
Excellent/Good	35	47.30	2.04	1.23-3.40	0.006			
Knowledge of English (n=897)								
None/Insufficient	78	28.78	1	-				
Sufficient/Fair	154	33.41	1.24	0.89-1.72	0.195			
Excellent/Good	65	39.63	1.62	1.08-2.44	0.020			
Participation in hospital research group								
No	196	27.45	1	-				
Yes	78	57.35	3.55	2.44-5.18	<0.001	1.54	0.93-2.54	0.091
Participation in at least one specific course on hospital research								
No	200	27.14	1	-				
Yes	74	65.49	5.09	3.35-7.76	<0.001	3.08	1.77-5.36	<0.001
Participation in other courses on research outside your hospital								
No	249	30.97	1	-				
Yes	25	54.35	2.65	1.46-4.83	<0.001			

Note: ±A Post-graduate Diploma (after a Bachelor's Degree); †A Post-graduate Diploma (after a Master's Degree).
