# Supplementary material for: Participation of nurses and allied health professionals in research activities: a survey in an academic tertiary pediatric hospital
Source: BMC Nurs. 2022 Jun 21;21:159. doi: 10.1186/s12912-022-00922-1 (PMC9210609; doi:10.1186/s12912-022-00922-1)
Supplement: Supplementary file 3 — Additional file 3: Supplemental Table 3. Descriptive variables of thesample and association with writing scientific papers: univariate andmultivariable logistic analysis. [file 12912_2022_922_MOESM3_ESM.pdf]

Supplemental Table 3. Descriptive variables of the sample and association with writing scientific papers: univariate and multivariable logistic analyses

	Scientific Publications		Univariate Analysis			Multivariate analysis		
	**n**	**%**	**OR**	**95%CI**	**P-value**	**OR**	**95%CI**	**P-value**
Gender								
Females	69	9.56	1	-				
Males	25	15.82	1.78	1.09-2.92	0.022			
Age in years								
22-29	19	9.27	1	-				
30-39	34	13.77	1.56	0.86-2.83	0.14			
40-49	18	10.59	0.96	0.51-1.81	0.43			
≥50	23	8.91	0.10	0.06-0.16	0.13			
Professional Qualification								
Registered Nurse	30	8.38	1	-				
Registered Pediatric Nurse	21	5.66	0.656	0.37-1.17	0.153	1.07	0.51-2.26	0.856
Allied Health Professional	43	28.48	4.35	2.60-7.28	<0.001	5.66	2.76-11.62	<0.001
Professional role								
Staff	66	8.52	1	-				
Manager	19	24.36	3.46	1.95-6.15	<0.001	2.15	0.88-5.28	0.094
Clinical expert	4	26.67	3.91	1.21-12.60	0.023	2.37	0.46-12.27	0.305
Fellow	5	41.67	7.67	2.37-24.85	0.001	0.78	0.15-4.00	0.767
Hospital employee								
No	15	10.71	1	-				
Yes	79	10.68	0.99	0.55-1.78	0.989			
Hospital center								
Sub-intensive neurorehabilitation, specialist medical and surgical	10	5.29	1	-				
Main building	66	11.19	2.25	1.13-4.48	0.02	2.69	1.07-6.77	0.036
Research laboratories and outpatients	15	20.00	4.48	1.91-10.49	0.001	5.35	1.58-18.07	0.007
Neurorehabilitation	3	12	2.44	0.62-9.55	0.200	0.49	0.04-5.48	0.563
Work experience in hospital (years) (n=880)								
≤4	24	10.57	1	-				
5-9	18	12.00	1.15	0.60-2.20	0.68			
10-19	26	12.50	1.21	0.67-2.18	0.53			
20-29	11	11.83	1.13	0.53-2.42	0.74			
≥30	15	7.43	0.68	0.35-1.33	0.26			
Education level (n=896)								
Other (Regional Diploma, University Diploma, etc.)	20	6.62	1	-				
Bachelor's Degree	74	12.80	2.07	1.24-3.46	0.006	1.40	0.63-3.11	0.403
Education for manager roles								
None	61	8.58	1	-				
Regional qualifying course for manager roles	10	21.28	2.88	1.37-6.07	0.005			
Master in Management	23	18.85	2.48	1.46-4.18	0.001			
Education for executive roles								
No	57	7.55	1	-				
Master of Science in Nursing	35	32.71	5.95	3.66-9.68	<0.001			
Director of Nursing Services	0	0	1	omitted				
Both	2	22.22	3.50	0.71-17.23	0.12			
Post-graduate education								
No	45	7.10	1	-				
Master ±	41	17.52	2.78	1.77-4.37	<0.001	2.58	1.43-4.63	0.002
Advanced Master†	6	75	39.27	7.7-200.16	<0.001	19.12	0.86-425.9	0.062

Post-graduate Courses or Regional specializations								
No	68	9.12	1	-				
Yes	25	21.01	2.65	1.59-4.40	<0.001	1.55	0.72-3.32	0.258
PhD (or PhD student)	4	0.43	1	-	<0.001			
Reading scientific journals (n=882)								
Frequency								
No	6	3.55	1	-				
Yes, occasionally	21	7.47	2.19	0.87-5.55	0.097	1.71	0.58-5.03	0.332
Yes, when I have to search for something	29	9.83	2.96	1.20-7.29	0.018	1.47	0.50-4.31	0.482
Yes, regularly	37	33.33	13.58	5.49-33.59	<0.001	5.51	1.79-17.00	0.003
Types of journals								
Italian	24	40.68	1	-				
International	24	4.46	3.84	2.21-6.65	<0.001			
Both	46	16.25	2.28	1.68-3.09	<0.001			
Knowledge of Epidemiology (n=897)								
None/Insufficient	23	8.36	1	-				
Sufficient/Fair	43	8.96	1.08	0.63-1.83	0.781			
Excellent/Good	28	22.40	3.16	1.74-5.76	<0.001			
Knowledge of Statistics (n=897)								
None/Insufficient	20	5.70	1	-				
Sufficient/Fair	51	11.21	2.09	1.22-3.57	0.007			
Excellent/Good	23	31.08	7.46	3.82-14.55	<0.001			
Knowledge of English (n=897)								
None/Insufficient	11	4.20	1	-				
Sufficient/Fair	54	11.89	3.08	1.58-6.00	0.001	1.95	0.84-4.53	0.121
Excellent/Good	29	17.68	4.90	2.37-10.12	<0.001	1.66	0.59-4.64	0.332
Participation in at least one								
Participation in hospital research group								
No	44	6.16	1	-				
Yes	39	28.68	6.12	3.78-9.90	<0.001	3.14	1.50-6.59	0.002
Participation in at least one specific course on research in hospital								
No	17	36.96	1	-				
Yes	66	8.21	2.1	3.42-12.55	<0.001	1.69	0.76-3.73	0.197
Participation in other courses on research outside the hospital								
No	66	8.21	1	-				
Yes	17	36.96	6.55	3.42-12.55	<0.001	2.42	0.94-6.23	0.067

Note: ±A Post-graduate Diploma (after a Bachelor's Degree); †A Post-graduate Diploma (after a Master's Degree).
