# Supplementary figures and images for: Participation of nurses and allied health professionals in research activities: a survey in an academic tertiary pediatric hospital
Source: BMC Nurs. 2022 Jun 21;21:159. doi: 10.1186/s12912-022-00922-1 (PMC9210609; doi:10.1186/s12912-022-00922-1)

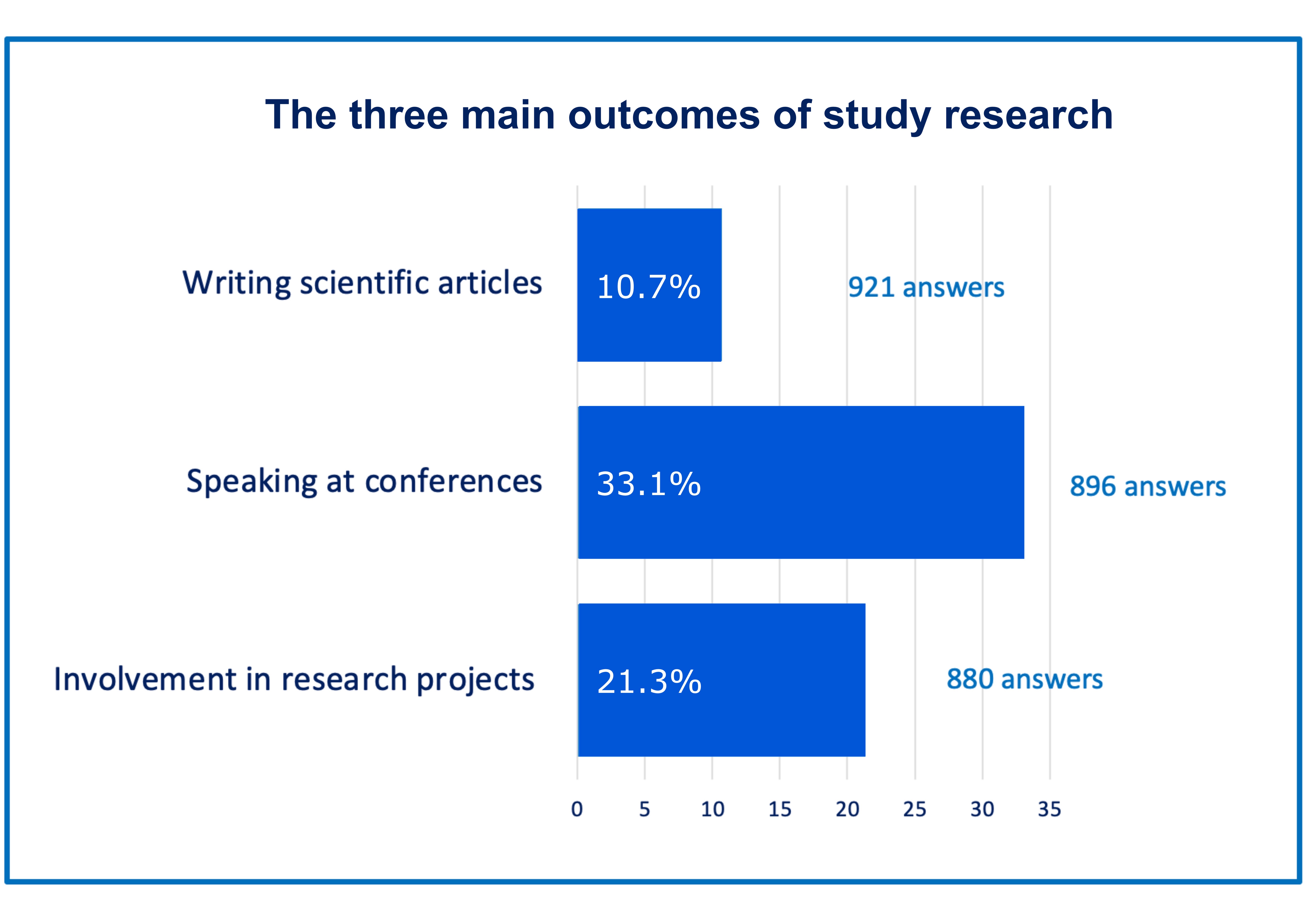

Supplement: Supplementary file 4 — Additional file 4: Supplemental Figure 1. The three main outcomes ofstudy research. [file 12912_2022_922_MOESM4_ESM.jpg]
